# Risk of Hyperglycemia and Diabetes after Early-Life Famine Exposure: A Cross-Sectional Survey in Northeastern China

**DOI:** 10.3390/ijerph15061125

**Published:** 2018-05-31

**Authors:** Yangyu Zhang, Xinyu Liu, Mohan Wang, Yan Song, Lili Zhang, Yueyue You, Yingying Su, Yingyu Liu, Changgui Kou

**Affiliations:** Department of Epidemiology and Biostatistics, School of Public Health, Jilin University, Changchun 130021, China; yyzhang15@mails.jlu.edu.cn (Y.Z.); liuxy16@mails.jlu.edu.cn (X.L.); wangmh16@mails.jlu.edu.cn (M.W.); songyan16@mails.jlu.edu.cn (Y.S.); zhangll16@mails.jlu.edu.cn (L.Z.); youyy15@mails.jlu.edu.cn (Y.Y.); suyy14@mails.jlu.edu.cn (Y.S.); liuyy15@mails.jlu.edu.cn (Y.L.)

**Keywords:** Chinese famine, malnutrition, hyperglycemia, diabetes, sex difference

## Abstract

Previous studies suggested that malnutrition during early life may play an essential role in later outcomes and disease risk in adulthood. We aimed to investigate the risks of hyperglycemia and diabetes 50 years after early-life famine exposure in a Northeastern Chinese population. We used the data from 5690 adults born between 1956 and 1965 in selected communities from a 2012 cross-sectional study. The early-childhood exposure cohort showed an increased risk of hyperglycemia compared with the unexposed cohort in the female population (odds ratio (OR) 1.46; 95% confidence interval (CI) 1.04, 2.06). The increased risk of diabetes in the early-childhood and fetal exposure cohorts was 37.0% (95% CI 1.05–1.79) and 50% (95% CI 1.15–1.96), respectively. For women, the risk of diabetes was more pronounced in the fetal-exposed cohort (OR 1.82; 95% CI 1.26–2.63) than in the early-childhood cohort (OR 1.57; 95% CI 1.08–2.26). Early-life exposure to famine increased the risk of diabetes. Furthermore, early-childhood exposure to famine might increase the risk of hyperglycemia in women. A policy for preventing early life malnutrition should be drafted by the government to prevent hyperglycemia and diabetes in adulthood.

## 1. Introduction

The thrifty phenotype hypothesis was postulated to explain the associations between poor fetal and infant condition and increased risk of type 2 diabetes (T2D) due to the influences of malnutrition in early life that leads to irreversible changes in glucose-insulin metabolism [1]. A general approach used to validate this hypothesis in human beings involves assessing the influence of a natural disaster, such as a famine, especially when it is consistent with the gestation or early-childhood periods, so that the prevalence of disease in these subjects during adulthoods can be observed. The first evidence of a direct link between malnutrition during gestation with decreased glucose tolerance in humans was found due to the Dutch famine in 1998 [2]. However, due to the ethical issues, the related research that verifies this hypothesis in humans is scarce.

The Chinese Great Famine began in 1959 and ended in 1961, being one of the largest disasters of human history. Almost all people living in mainland China were impacted, and the total death toll caused by the Chinese famine was approximately 15–30 million [3,4,5]. The influence of early-life malnutrition on adulthood health has already been reported in different countries [6,7,8,9], and an increasing number of studies of Chinese populations have searched for the relationships between early-life exposure to famine and chronic diseases in later life. However, the results have been inconsistent [10,11,12]. The evidence for the effects of exposure to poor conditions in early life among people in Northeastern China is inadequate, especially for a follow-up time of about 50 years after exposure. Due to a decline in the secretion of insulin, insulin resistance, and an increase in body mass index (BMI), the prevalence of hyperglycemia and T2D increases with age [13,14,15]. This study was designed to determine the risk for hyperglycemia and diabetes 50 years after fetal and early-childhood exposure to the Chinese famine in a northeastern population.

## 2. Materials and Methods

### 2.1. Data Source

Data were collected from the Chronic Disease Survey that was conducted from June to August 2012 in Jilin Province. Jilin Province is located in Northeast China with a population of approximately 27.5 million inhabitants. We used multistage stratified cluster sampling to choose the target sample from community-dwelling residents aged 18–79 years in Jilin. More details about this survey have been previously described [16]. This study selected the subjects who were born between 1 January 1956, and 31 December 1965.

### 2.2. Ethics Statement

The protocol used for the Chronic Disease Survey was approved by the Health Bureau of Jilin Province (Reference number: 2012-10) and the Ethics Committee of the School of Public Health, Jilin University (Reference number: 2012-R-011). Informed consent was acquired from all participants.

### 2.3. Chinese Famine and Famine Cohort

The Chinese famine that occurred from 1959 to 1961, the largest one in human history, was undiscovered for a long time in the West, until researchers reviewed census data released by the central government in the 1980s. The researchers found that China lost up to 30 million people during the famine years [2,3,4,5]. The immediate cause of the famine was the Great Leap Forward campaign in 1958. Thousands of people’s communes organized all the rural households, leading to a sharp and sudden drop in food production during 1959–1961. At the same time, drought, excessive procurement by the government, and the sluggish response to the food shortage eroded incentives for grain production due to collectivization and resource diversion. In addition, massive industrialization also played a role in this famine [3,4,17].

Subjects were categorized into four exposure cohorts: early-childhood exposure, fetal exposure, transitional, and unexposed. The four cohorts were defined by the subjects’ dates of birth. Subjects that were born between 1 January 1956 and 31 December 1958 were classified as the early-childhood exposure cohort; subjects born between 1 January 1959 and 31 December 1961 were classified as the fetal-exposed cohort; subjects born between 1 January 1962 and 31 December 1962 were classified as the transitional cohort, as determining their exposure to famine was difficult; and subjects born between 1 January 1963 and 31 December 1965 were classified as unexposed. Such classification was used in previous Chinese famine studies [18,19]. The mean ages for subjects in the early-childhood exposure cohort, fetal exposure cohort, transitional cohort, and unexposed cohort were 54.5, 51.6, 49.5, and 47.7 years, respectively.

### 2.4. Data Measurement

Height, body weight, and waist circumference (WC) measurement were recorded in duplicate by professional trainers. All participants were required to remove shoes and wear light clothing. A mercury sphygmomanometer was used to measure blood pressure. After overnight fasting, fasting blood glucose (FBG) was measured before breakfast using a Bai Ankang fingertip blood glucose monitor (Bayer, Leverkusen, Germany) using fingertip blood samples. Following the same method, 2 h plasma glucose (2 hPG) was tested.

### 2.5. Assessment Criteria

The body mass index (BMI) is calculated using the ratio of body weight in kilograms to the squared body height in meters (kg/m^2^). According to the Chinese “Guideline for prevention and control of overweight and obesity in Chinese adults” [20], participants were defined as underweight with a BMI below18.5 kg/m^2^, normal with a BMI between 18.5 and 23.9 kg/m^2^, overweight with a BMI over 24 kg/m^2^, and obese with a BMI over 28 kg/m^2^. Participants who drank any kind of alcoholic beverage at least once per week over the past year before the survey were classified as alcohol consumers. Those who reported smoking at least 100 cigarettes in their lifetime and smoked either every day or some days, at the time of the survey, were defined as smokers. Subjects classified as exercising often were those who exercised more than three times a week; those who exercised one or two times per week were defined as occasionally exercising; and subjects who did not or seldom exercised were defined as never exercising. Fruit intake was divided into “often”, “occasional”, and “never”: those who ingested fruit more than three times a week were classified as often, those who ingested one or two times a week were defined as occasional; those who did not or seldomly ingested fruit were defined as never. The World Health Organization (WHO) criteria [21] were used to diagnose diabetes mellitus. An FBG of 7.0 mmol/L or higher was considered diabetes, and hyperglycemia was defined as having an FBG between 6.1 and 6.9 mmol/L. A subgroup reported a 2 h plasma glucose level, in which hyperglycemia was defined as a 2 hPG between 7.8 and 11.0 mmol/L and diabetes as a 2 hPG ≥ 11.1 mmol/L. Participants who had been diagnosed with diabetes or had previously used antidiabetic drugs were also considered diabetics.

### 2.6. Statistical Analyses

The demographics and clinical characteristics are presented as means (± SD) and proportions (%). Group differences were tested using analysis of variance (ANOVA) and chi-squared test. Logistic regression analyses were used to calculate odds ratios (ORs) and evaluate the associations between exposure (early childhood and fetal exposure to famine), potential confounders (BMI, sex, region, education, smoking status, drinking status, physical activity, and fruit intake) and outcomes (hyperglycemia and diabetes). As the unexposed group was younger than the fetal group, to adjust for the potential age effect, we combined unexposed and early-childhood exposure as reference groups in the logistic regression to calculate the OR for diabetes among those in the fetal exposure group, similarly to the previous studies [19,22]. Moreover, several previous reports discovered that adult outcomes were associated with sex and BMI, so we also stratified our analyses on sex and BMI [6,7,23]. All statistical analyses were performed using the IBM SPSS 21.0 software (IBM SPSS, IBM Corp, Armonk, NY, USA). A *p*-value of 0.05 was considered statistically significant.

## 3. Results

### 3.1. Subject Characteristics

Basic characteristics of the subjects are shown in Table 1. A total of 5960 subjects were studied, including 2637 (46.3%) men and 3053 (53.7%) women. Among these subjects, 1442 (25.3%) were exposed to the Chinese famine during fetal life, whereas 1582 (27.8%) were exposed during early-childhood, and 1986 (34.9%) unexposed subjects were defined as the control group. The proportions of smokers were different in the four groups, but alcohol consumption was found to be equally distributed among cohorts. Educational level, physical activity frequency, and fruit intake were found to be unequally distributed among cohorts.

### 3.2. Hyperglycemia

Table 2 presents the prevalence rates of hyperglycemia among the four cohorts. The prevalence of hyperglycemia in the early-childhood exposure, fetal exposure, transitional, and unexposed groups was 10.0, 10.3, 9.4, and 9.1%, respectively. The risk of hyperglycemia in either of three cohorts was not significantly higher than the unexposed cohort, even after adjusting for BMI, sex, region, smoking, drinking, physical activity, and fruit intake. When we conducted stratified analysis by sex, we found the prevalence rates of hyperglycemia among the four groups in men were all higher than in women. However, the hyperglycemia risk for women exposed to famine in early childhood was significantly higher (OR 1.46; 95% CI 1.04, 2.06) than for the non-exposed cohort, even after adjusting for BMI (OR 1.46; 95% CI 1.03, 2.05) and additional adjustments for sex, region, education, smoking status, drinking status, physical activity, and fruit intake (OR 1.55; 95% CI 1.10, 2.19). However, this difference was not observed in the male subjects.

### 3.3. Diabetes

The results evaluating the risk of diabetes later in life after famine exposure in early life are shown in Table 3. The prevalence of diabetes in the early-childhood exposure, fetal exposure, transitional, and non-exposed cohort was 7.8, 8.5, 7.1, and 5.8%, respectively. Compared with the control group, the risk for diabetes in the early-childhood and fetal exposure cohorts increased by 37.0% (95% CI 1.05–1.79) and 50% (95% CI 1.15–1.96), respectively. After adjusting BMI, the risk of diabetes in the early-childhood and fetal famine-exposure cohorts was still significantly different from the unexposed cohort, with the risk increasing by 42% (95% CI 1.08–1.85), 51% (95% CI 1.15–1.98), respectively. After additionally adjusting for sex, region, smoking, drinking, physical activity, and fruit intake, the early-childhood exposure cohort showed no significantly higher risk of diabetes; however, the risk in the fetal exposure cohort still increased by 40% (95% CI 1.06–1.85). Using unexposed group and early-childhood exposure as reference, the fetal exposure group had an OR of 1.29 (95% CI 1.03–1.62) for diabetes. After adjusting for BMI, the fetal exposure cohort still showed significantly higher risk of diabetes, with the risk increasing by 28% (95% CI 1.01–1.61). The odds ratio of being diabetic in women was more pronounced in the fetal exposure cohort (OR 1.82; 95% CI 1.26–2.63) than in the early-childhood exposure cohort (OR 1.57; 95% CI 1.08–2.26). After adjusting for the potential confounder BMI in women, the risk of diabetes in the fetal exposure cohort was still higher (OR 1.88; 95% CI 1.29–2.73) than the early-childhood exposure cohort (OR 1.59; 95% CI 1.09–2.31). After additionally adjusting for other confounders, the result changed, and only the fetal exposure group had a significantly higher risk, with an odds ratio of 1.67 (95% CI 1.12–2.49). Moreover, in women, we observed that the fetal exposure group (OR 1.54; 95% CI 1.10–2.15) significantly increased the risk of diabetes, compared with the combined group (non-exposure and early-childhood exposure), even after adjusting for the confounding factors. For the men, all the cohorts or models presented no significant differences.

According to the BMI value, we divided the individuals into three groups: underweight or normal, overweight, and obese. Table 4 shows the risk of diabetes after famine exposure in the three groups. After adjusting for the confounding factors, the underweight or normal individuals who were exposed in early childhood to famine had a significantly higher risk of diabetes than the control group (OR 1.80; 95% CI 1.06–3.06). In overweight women, the risk of diabetes in the fetal exposure cohort was more evident (OR 2.35; 95% CI 1.26–4.41) even after adjusting for region, education, smoking status, drinking status, physical activity, and fruit intake. For the men, all the cohorts presented no significant differences, as shown in Table 4.

## 4. Discussion

From a sample of Northeastern Chinese adults in our study, we found a conspicuous association between famine exposure during the early-childhood period and an increased risk of hyperglycemia in adult women. Compared with the unexposed cohort, fetal undernutrition was associated with significant increases in the prevalence of diabetes. This association was more pronounced in women, even after adjusting for the confounding factors. No consistent association was observed in men. The fetal exposure overweight women had a significantly higher risk of diabetes than the unexposed cohort. Previous studies showed that nutrition during infancy may significantly impact health in later life and in the risk of diseases [6,24]. Childhood nutritional condition, especially during the first few months of postnatal life, is a crucial factor in the propensity to develop disease in adulthood [25]. Our study found a significantly increased risk of hyperglycemia in the early-childhood exposure cohort only in women, which indicates that malnutrition is an extremely important risk factor for adulthood hyperglycemia for women during infancy.

Our findings are consistent with previous studies that suggested that early life malnutrition is associated with type 2 diabetes risk in later life [26,27,28]. In our study, the risks of diabetes in the early-childhood exposure and fetal exposure cohorts were all significant. After adjusting for the confounding factors in model 2 (Table 3), only the fetal exposure cohort showed differences from the unexposed cohort. The results of stratified analysis by sex showed that early childhood and fetal exposure to famine resulted in an excess risk for diabetes in women. The experimental studies showed that the mechanisms of fetal programming on insulin resistance and insulin secretion may be sex-specific, which may be related to the interpretation of sex differences in glucose tolerance [29].

The association between prenatal undernutrition and glucose tolerance in adulthood might be explained by several mechanisms. Animal experiments found that pancreatic development was impacted by poor conditions during gestation, which led to the functional impairment of β-cells and a subsequent insulin deficiency [30]. The majority of human experiments indicated that insulin resistance may play a key role in the occurrence of diabetes [31,32] and this possible explanation was supported by a Dutch cohort [33]. A large amount of evidence demonstrated that skeletal muscle is an essential site for the programming of insulin resistance. Muscle is a crucial site of glucose absorption and a low ponderal index was found to be related to the metabolic changes of adult skeletal muscle. Another possible cause is that individuals who endure survival stress due to poor nutrition might alter the set point of their hypothalamic–pituitary–adrenal (HPA) axis, which may result in the long term alteration of the secretion of the neuroendocrine mediators of the stress response, and subsequently suffer from cardiovascular and metabolic disease in adulthood [34,35].

Being overweight may partly represent a nutritional “rich” environment. In our study, overweight women in the fetal exposure cohort had a high risk of diabetes. Similar results were found in other studies [6,27]. The rich nutrition status mismatch with the extremely poor fetal environment condition might increase the risk of diabetes in later life. According to our study, the associations between fetal exposure to famine and risk of diabetes were stronger among women who became overweight in later life.

The Great Chinese Famine between 1959 and 1961 was a three-year period of extreme food shortage. The role of undernutrition during early life for future health in adults has been previously evaluated in China [27,36], but Northeastern China, having a distinct biogeoclimate and lifestyle, has been seldomly investigated. Our study, with population specificity, is representative of the inhabitants of Jilin Province in Northeastern China. Animal experiments demonstrated the effects of prenatal undernutrition on glucose tolerance that increased with age. Rats malnourished during gestation showed a decrease in glucose tolerance between 3 and both 12 and 15 months of age [26]. This study, using a unique population more than 50 years after the Great Chinese Famine in Jilin region, consequently, was significant and needed.

This study was part of the Jilin Provincial Chronic Disease Survey, which was a large-scale, cross-sectional epidemiology study. The community-dwelling residents were recruited using a multistage stratified cluster sampling method. Therefore, we believe that the study subjects are representative of the residents of Jilin Province. Moreover, due to the exclusion of subjects born in the transitional period, there was no late gestational overlap with famine in the unexposed group. Our study has several limitations. The first limitation was that we did not have data for birth weight, which is the most commonly used index for fetal undernutrition [37]. Secondly, we also did not have data about the exact severity of mother and infant malnutrition. Thirdly, as the Chinese famine lasted for three years (1959–1961), individuals who were exposed to famine during the fetal period might also have been exposed during infancy period. Despite these limitations, our study was conducted at an appropriate time after the famine period, and the follow-up was long enough after the famine to establish relationships to outcomes that are directly related to adult diabetes.

People generally believe that malnutrition only occurs in developing countries and regions, but according to the Global Nutrition Report in 2016 [38], nearly half of the countries in the world were suffering from poor nutrition and obesity, and malnutrition in early life was a major global public health issue. Moreover, malnutrition in children was widespread in China, especially in seriously impoverished area. Studies [39,40,41] showed that poor economic conditions and low levels of nutritional awareness are the important causes of poor nutrition in early life. Therefore, we hope that this study about the significance of early-life nutrition will help draw more attention to this problem. The government must formulate policies to strengthen pregnancy and early-childhood nutritional surveillance and improve nutritional status. The government also should conduct educational interventions to raise the public awareness of rational nutrition. For countries and regions still suffering from famine, including nutritional food supplementation projects for pregnant women and infants is essential.

## 5. Conclusions

In conclusion, we found that exposure to severe famine in fetal life increased the risk of diabetes in later life. The women who were exposed to famine in their early childhood had an increased susceptibility to hyperglycemia. Overweight women who experienced fetal exposure to famine had a higher risk of diabetes. Together with previous studies, our study emphasizes that the early-life environment is critical for the risk of hyperglycemia and diabetes in adulthood. A policy for preventing malnutrition in early life should be formulated by the government to prevent hyperglycemia and diabetes in later life.

## Figures and Tables

**Table 1:** Baseline characteristic of subjects in the four cohorts based on birth date.

Characteristic	Early-Childhood Exposure, *n* (%)/Mean ± SD	Fetal Exposure, *n* (%)/Mean ± SD	Transitional Period, *n* (%)/Mean ± SD	Unexposed, *n* (%)/Mean ± SD	*p*-Value
Number of subjects	1582	1442	680	1986	
Gender, male	729 (46.1)	688 (47.7)	335 (49.3)	885 (44.6)	0.112
Age, years	54.52 ± 0.93	51.58 ± 0.92	49.50 ± 0.50	47.66 ± 0.96	<0.001
Urban	749 (47.3)	731 (50.7)	325 (47.8)	969 (48.8)	0.298
**Education**					<0.001
Primary school or below	516 (32.6)	368 (25.5)	168 (24.7)	452 (22.8)	
Junior school	389 (24.6)	334 (23.2)	167 (24.6)	581 (29.3)	
Senior school	538 (34.0)	606 (42.0)	273 (40.1)	645 (32.5)	
College or above	139 (8.8)	134 (9.3)	72 (10.6)	308 (15.5)	
Weight, kg	64.55 ± 10.80	65.71 ± 11.14	65.67 ± 10.89	65.94 ± 11.42	0.002
Height, cm	162.24 ± 8.12	162.68 ± 8.23	162.82 ± 7.89	162.98 ± 8.05	0.060
Waist circumference, cm	84.06 ± 9.74	84.14 ± 10.08	83.71 ± 9.51	83.48 ± 10.22	0.196
BMI, kg/m^2^	24.48 ± 3.46	24.78 ± 3.45	24.71 ± 3.31	24.77 ± 3.55	0.062
Hyperglycemia	158 (10.0)	149 (10.3)	64 (9.4)	180 (9.1)	0.617
Type 2 diabetes	123 (7.8)	122 (8.5)	48 (7.1)	115 (5.8)	0.017
Smoking	533 (33.7)	474 (32.9)	252 (37.1)	599 (30.2)	0.006
Alcohol consumption	494 (31.2)	443 (30.7)	223 (32.8)	675 (34.0)	0.162
**Physical activity**					<0.001
Often	597 (37.7)	449 (31.1)	203 (29.9)	557 (28.0)	
Occasionally	266 (16.8)	264 (18.3)	140 (20.6)	450 (22.7)	
Never	719 (45.4)	729 (50.6)	337 (49.6)	979 (49.3)	
**Fruit intake**					<0.001
Often	780 (49.3)	691 (47.9)	304 (44.7)	1,013 (51.0)	
Occasionally	409 (25.9)	389 (27.0)	199 (29.3)	608 (30.6)	
Never	393 (24.8)	362 (25.1)	177 (26.0)	365 (18.4)	

BMI: body mass index.

**Table 2:** Association of famine exposure with risk of hyperglycemia in Northeastern Chinese population.

Gender and Model	Early-Childhood Exposure	Fetal Exposure	Transitional	Unexposed
Both genders				
Prevalence, *n* (%)	158 (10.0)	149 (10.3)	64 (9.4)	180 (9.1)
Crude	1.13 (0.89–1.39)	1.16 (0.92–1.45)	1.04 (0.77–1.41)	Reference
Model 1 ^1^	1.13 (0.90–1.41)	1.15 (0.92–1.45)	1.05 (0.78–1.42)	Reference
Model 2 ^2^	1.17 (0.93–1.47)	1.15 (0.91,1.45)	1.02 (0.75–1.38)	Reference
Male				
Prevalence, *n* (%)	83 (11.4)	89 (12.9)	42 (12.5)	112 (12.7)
Crude	0.89 (0.66–1.20)	1.03 (0.76–1.38)	0.99 (0.68–1.45)	Reference
Model 1 ^1^	0.91 (0.67–1.24)	1.03 (0.76–1.39)	0.98 (0.67–1.43)	Reference
Model 2 ^3^	0.93 (0.68–1.27)	1.01 (0.75–1.37)	0.96 (0.66–1.42)	Reference
Female				
Prevalence, *n* (%)	75 (8.8)	60 (8.0)	22 (6.4)	68 (6.2)
Crude	1.46 (1.04–2.06)	1.31 (0.92–1.88)	1.04 (0.63–1.70)	Reference
Model 1 ^1^	1.46 (1.03–2.05)	1.30 (0.90–1.86)	1.06 (0.64–1.75)	Reference
Model 2 ^3^	1.55 (1.10–2.19)	1.35 (0.94–1.94)	1.07 (0.65–1.76)	Reference

^1^ Adjustment for BMI; ^2^ Adjustment for BMI, gender, region, education, smoking status, drinking status, physical activity, and fruit intake; ^3^ Adjustment for BMI, region, education, smoking status, drinking status, physical activity, and fruit intake.

**Table 3:** Associations of famine exposure with the risk of diabetes in a Northeastern Chinese population.

Gender and Model	Early-Childhood Exposure	Fetal Exposure	Transitional	Unexposed	Fetal Exposure vs. Early-Childhood Exposure and Unexposed Combined
Both genders					
Prevalence, *n* (%)	123 (7.8)	122 (8.5)	48 (7.1)	115 (5.8)	
Crude	1.37 (1.05–1.79)	1.50 (1.15–1.96)	1.24 (0.87–1.75)	Reference	1.29 (1.03–1.62)
Model 1 ^1^	1.42 (1.08–1.85)	1.51 (1.15–1.98)	1.29 (0.91–1.84)	Reference	1.28 (1.01–1.61)
Model 2 ^2^	1.22 (0.92–1.61)	1.40 (1.06–1.85)	1.16 (0.80–1.67)	Reference	1.27 (1.00–1.62)
Male					
Prevalence, *n* (%)	57 (7.8)	55 (8.0)	26 (7.8)	59 (6.7)	
Crude	1.19 (0.81–1.73)	1.22 (0.83–1.78)	1.18 (0.73–1.90)	Reference	1.12 (0.80–1.57)
Model 1 ^1^	1.27 (0.86–1.87)	1.18 (0.80–1.75)	1.20 (0.74–1.94)	Reference	1.06 (0.75–1.50)
Model 2 ^3^	1.18 (0.80–1.76)	1.10 (0.74–1.65)	1.11 (0.68–1.83)	Reference	1.03 (0.73–1.47)
Female					
Prevalence, *n* (%)	66 (7.7)	67 (8.9)	22 (6.4)	56 (5.1)	
Crude	1.57 (1.08–2.26)	1.82 (1.26–2.63)	1.27 (0.76–2.11)	Reference	1.46 (1.07–2.00)
Model 1 ^1^	1.59 (1.09–2.31)	1.88 (1.29–2.73)	1.36 (0.81–2.27)	Reference	1.49 (1.09–2.05)
Model 2 ^3^	1.22 (0.81–1.82)	1.67 (1.12–2.49)	1.18 (0.69–2.03)	Reference	1.54 (1.10–2.15)

^1^ Adjustment for BMI; ^2^ Adjustment for BMI, gender, region, education, smoking status, drinking status, physical activity, and fruit intake; ^3^ Adjustment for BMI, region, education, smoking status, drinking status, physical activity, and fruit intake.

**Table 4:** Associations between the Chinese famine exposure and risk of diabetes stratified by BMI after adjusting for confounders.

Gender and Body Characteristic	Early-Childhood Exposure	Fetal Exposure	Transitional	Unexposed
Both genders ^1^				
Underweight or normal	1.80 (1.06–3.06)	1.34 (0.75–2.40)	1.75 (0.90–3.39)	Reference
Overweight	1.02 (0.66–1.59)	1.52 (0.99–2.31)	1.39 (0.82–2.35)	Reference
Obese	1.09 (0.65–1.84)	1.30 (0.79–2.15)	0.42 (0.17–1.03)	Reference
Male ^2^				
Underweight or normal	1.98 (0.86–4.56)	1.50 (0.62–3.64)	2.14 (0.82–5.61)	Reference
Overweight	1.04 (0.58–1.87)	0.87 (0.47–1.61)	1.16 (0.56–2.41)	Reference
Obese	0.98 (0.45–2.14)	1.33 (0.66–2.69)	0.40 (0.13–1.26)	Reference
Female ^2^				
Underweight or normal	1.58 (0.77–3.21)	1.19 (0.54–2.63)	1.35 (0.51–3.55)	Reference
Overweight	0.96 (0.48–1.91)	2.35 (1.26–4.41)	1.67 (0.76–3.66)	Reference
Obese	1.16 (0.57–2.37)	1.33 (0.64–2.75)	0.35 (0.08–1.65)	Reference

^1^ Adjustment for gender, region, education, smoking status, drinking status, physical activity, and fruit intake; ^2^ Adjustment for region, education, smoking status, drinking status, physical activity, and fruit intake.
